# Metabolic network as an objective biomarker in monitoring deep brain stimulation for Parkinson’s disease: a longitudinal study

**DOI:** 10.1186/s13550-020-00722-1

**Published:** 2020-10-29

**Authors:** Jingjie Ge, Min Wang, Wei Lin, Ping Wu, Yihui Guan, Huiwei Zhang, Zhemin Huang, Likun Yang, Chuantao Zuo, Jiehui Jiang, Axel Rominger, Kuangyu Shi

**Affiliations:** 1 PET Center, Huashan Hospital, Fudan University, 518 East Wuzhong Road, Shanghai, 200235 China; 2 Shanghai Institute for Advanced Communication and Data Science, Shanghai University, 99 Shangda Road, Shanghai, 200444 China; 3 Department of Neurosurgery, 904 Hospital of PLA, Wuxi, China; 4 Institute of Functional and Molecular Medical Imaging, Fudan University, Shanghai, China; 5 Key Laboratory of Specialty Fiber Optics and Optical Access Networks, Joint International Research Laboratory of Specialty Fiber Optics and Advanced Communication, Shanghai University, Shanghai, China; 6 Department of Nuclear Medicine, Inselspital, Bern University Hospital, University of Bern, Bern, Switzerland; 7 Department of Informatics, Technical University of Munich, Munich, Germany

**Keywords:** PD-related metabolic network pattern, Spatial covariance analysis, Parkinson’s disease, Deep brain stimulation, Graph theory

## Abstract

**Background:**

With the advance of subthalamic nucleus (STN) deep brain stimulation (DBS) in the treatment of Parkinson’s disease (PD), it is desired to identify objective criteria for the monitoring of the therapy outcome. This paper explores the feasibility of metabolic network derived from positron emission tomography (PET) with ^18^F-fluorodeoxyglucose in monitoring the STN DBS treatment for PD.

**Methods:**

Age-matched 33 PD patients, 33 healthy controls (HCs), 9 PD patients with bilateral DBS surgery and 9 controls underwent ^18^F-FDG PET scans. The DBS patients were followed longitudinally to investigate the alternations of the PD-related metabolic covariance pattern (PDRP) expressions.

**Results:**

The PDRP expression was abnormally elevated in PD patients compared with HCs (*P* < 0.001). For DBS patients, a significant decrease in the Unified Parkinson’s Disease Rating Scale (UPDRS, *P* = 0.001) and PDRP expression (*P* = 0.004) was observed 3 months after STN DBS treatment, while a rollback was observed in both UPDRS and PDRP expressions (both *P* < 0.01) 12 months after treatment. The changes in PDRP expression mediated by STN DBS were generally in line with UPDRS improvement. The graphical network analysis shows increased connections at 3 months and a return at 12 months confirmed by small-worldness coefficient.

**Conclusions:**

The preliminary results demonstrate the potential of metabolic network expression as complimentary objective biomarker for the assessment and monitoring of STN DBS treatment in PD patients.

*Clinical Trial Registration* ChiCTR-DOC-16008645. http://www.chictr.org.cn/showproj.aspx?proj=13865.

## Introduction

Since it was introduced in the early 1990s, deep brain stimulation (DBS) has become the second milestone in the treatment of Parkinson’s disease (PD) after dopamine replacement therapy. High-frequency stimulation with DBS targeting the subthalamic nucleus (STN) has proven to effectively improve motor dysfunction in PD patients. Prospective multicenter studies have reported that the Unified Parkinson's Disease Rating Scale (UPDRS) score 6 months after the first lead implantation was reduced by 23% compared to baseline in off-medication conditions [1], with the most significant amelioration occurring in tremor, rigidity and bradykinesia [2]. However, the UPDRS is a subjective criteria, which varies by different examiners [3]. It is also influenced by the background and status of patients such as educational level, age and cultural differences [4]. This becomes more difficult for the assessment of DBS treatment, which may be biased from psychological and physical fatigue, depression, and side effects caused by drugs of patients [5]. The possible deviation may hamper the optimization of DBS treatment. Therefore, an objective biomarker is desired for the assessment and monitoring of DBS treatment.

Functional imaging techniques such as metabolic brain imaging with ^18^F-fluorodeoxyglucose (FDG) positron emission tomography (PET) could provide an objective way to map and quantify changes in local neural activity associated with brain diseases. In particular, abnormal PD-related metabolic covariance patterns (PDRP) have typically been identified by spatial covariance analysis, whose expression yields the better discrimination of PD patients at an individual level. It has been reported that PDRP activities could accurately discriminate PD patients not only from controls but also from individuals with atypical Parkinsonism (i.e., multiple system atrophy or progressive supranuclear palsy) [6]. Moreover, the increased expression of PDRP revealed consistent correlation with motor manifestations [7] and was useful for detecting the prodromal stage of parkinsonism (i.e., rapid eye movement sleep behavior disorder) by predicting phenoconversion toward subsequent development [8, 9]. However, few studies explored whether the PDRP expression could be used to measure the therapy outcome of STN DBS for PD at an individual level.

PDRP activities have also been shown to characterize alterations in cerebral metabolic function linking the treatment outcomes in PD patients. Indeed, inhibited PDRP activities were observed to be induced by STN DBS as demonstrated by decreased metabolism in the putamen/globus pallidus, sensorimotor cortex and cerebellar vermis, covarying with increased metabolism in the precuneus, which indicated a potential correlation between ameliorated motor symptoms and the corrected function underlying the abnormally overexpressed circuits of the cortico-striato-pallido-thalamo-cortical (CSPTC) pathway for motor control in PD patients [10]. It is notable that these investigations directly compared STN DBS “ON” and “OFF” states at the same time point after electrode implantation, critically reflecting the immediate interference of external local stimuli on the whole brain [5, 10]. The immediate interference of DBS may be effected by the after discharge of neurostimulation, and the observation of DBS “OFF” is controversial with DBS long-term potentiation [11].

Therefore, the purpose of the current study was to examine whether the PDRP expression could be as a potential objective biomarker for monitoring STN DBS treatment outcome, for which we conducted a longitudinal follow-up study comparing ^18^F-FDG PET imaging in the same patients at different time points (baseline, 3 months and 12 months) before and after treatment. In addition to examine the feasibility of PDRP expression, this study also investigated the underlying connectome to elucidate the efficiency of PDRP. The connections can provide an in-depth insight into disease-associated abnormalities and therapy-induced effects within ROIs of PDRP [12–14]. Graph theory was applied to investigate the topology of disease networks within PDRP [15], and exaggerated small-world properties were revealed.

## Methods

### Subjects

We investigated two independent cohorts of subjects with ^18^F-FDG PET images in this study (Table 1). Cohort I comprised 33 PD patients (PD1) and 33 age-/gender-matched healthy controls (HC1) recruited from Huashan Hospital, Fudan University, Shanghai, China. The scans from cohort I were used for the identification of region-of-interest (ROI)-based PDRP topography. Cohort II included 9 PD patients (PD2) and 9 age-/gender-matched healthy controls (HC2) recruited from 904 Hospital, Wuxi, China. Each PD patient from cohort II underwent bilateral STN DBS implantation. Four of the 9 patients were scanned 3 times with ^18^F-FDG PET images (preoperative baseline, 3-month post-operation and 12-month post-operation), while the other 5 patients were scanned twice (preoperative baseline and 3-month post-operation). Of the 5 subjects who did not complete the final follow-up, 2 relocated and 3 refused to participate in further scanning. Imaging data from cohort II were used to assess the effects of clinically effective STN DBS on the metabolic variation in PD patients.

**Table 1:** Demographic and clinical characteristics of healthy controls and PD patients

Cohort	Gender (M/F)	Age (years)	Duration of disease (years)	UPDRS			PDRP Z-score		
				Preoperative baseline	Post-operation		Preoperative baseline	Post-operation	
					3 months	12 months		3 months	12 months
*Cohort I*									
HC1	15/18	57.4 ± 10.5	–	–	–	–	0 ± 1.0	–	–
PD1	15/18	58.1 ± 10.3	4.3 ± 4.1	25.2 ± 14.4	–	–	2.74 ± 1.18	–	–
*Cohort II*									
HC2	4/5	61.7 ± 7.3	–	–	–	–	0 ± 0.52	–	–
*PD2 (patients underwent DBS)*									
1	F	50	8	49	21	60	3.01	0.11	1.83
2	M	66	19	62	53	69	1.61	− 0.18	0.17
3	M	62	10	51	10	37	2.42	1.97	2.28
4	F	72	5	50	12	31	1.52	− 0.40	0.39
5	M	53	17	39	30	–	4.21	2.84	–
6	F	54	13	52	31	–	2.89	2.77	–
7	F	77	7	51	24	–	3.71	2.53	–
8	F	69	4	58	56	–	2.69	2.11	–
9	M	65	6	35	10	–	0.72	0.26	–
Total	4/5	63.1 ± 9.2	9.9 ± 5.3	49.7 ± 8.4	27.4 ± 17.3	49.3 ± 18.2	2.53 ± 1.11	1.33 ± 1.35	1.17 ± 1.04

Data are given as mean ± standard deviation

*UPDRS* Unified Parkinson’s Disease Rating Scale

All PD patients enrolled in this study satisfied the diagnostic criteria according to the United Kingdom Parkinson’s Disease Society Brain Bank and showed no structural brain abnormalities (e.g., mass lesions or ischemia) on magnetic resonance imaging (MRI) scans [16]. Each PD patient was evaluated by the Unified Parkinson’s Disease Rating Scale (UPDRS) by a senior movement disorder specialist on the same day where ^18^F-FDG PET images were acquired, at least 12 h after the cessation of oral antiparkinsonian medication. For PD2 patients, PET images and clinical evaluation were conducted in the STN DBS “OFF” state, in the absence of STN stimulation at least 2 h prior to PET imaging at all time points [17]. The PD2 patients were at very advanced clinical stages compared to PD1 patients with longer disease durations (*P* = 0.001) and more severe motor symptoms (*P* < 0.001) (Table 1). All healthy control participants in this study underwent a neurological examination to rule out a history of neurological or psychiatric disorders. None of the patients or healthy subjects had any prior exposure to neuroleptic agents or drug use.

Ethical permission for the study procedures was obtained from the Institutional Review Boards at Huashan Hospital and 904 Hospital. This study was performed in compliance with the Code of Ethics of the World Medical Association (Declaration of Helsinki) and the standards established by the author’s local Institutional Review Boards and grant agencies. Written consent was obtained at each institution from each subject following a detailed explanation of the scanning procedures. Authors had access to information that could identify individual participants during or after data collection. All procedures performed in studies involving human participants were in accordance with the ethical standards of the institutional and/or national research committee and with the 1964 Declaration of Helsinki and its later amendments or comparable ethical standards.

### STN DBS

The 9 PD patients who underwent DBS surgery received bilateral electrodes (Model 3389, Medtronic, USA) implanted in the STN at 904 Hospital, Wuxi, China. The installation of a Leksell stereotaxic frame was performed under local anesthesia. Stereotactic MRI was used during surgery for both preoperative targeting and immediate post-operative verification, verifying correct electrode placement within the STN. Stimulation parameters (frequency, pulse width and voltage) were set by the programmed protocol 2 weeks after implantation to achieve the best control of symptoms without adverse side effects [18].

### PET images and preprocessing

In our study, all participants were asked to fast for at least 6 h but had free access to water prior to ^18^F-FDG PET scanning. All patients underwent scanning at least 12 h after the cessation of oral antiparkinsonian medications. Subjects from cohort I were scanned on a Siemens Biograph 64 PET/CT (Siemens, Erlangen, Germany) in three-dimensional mode. After a CT transmission scan for attenuation correction, a PET image was acquired over 45–55 min post-injection of ^18^F-FDG (8–10 mCi) and was reconstructed with the ordered subset expectation maximization method. Individuals from cohort II were scanned with a GE Advance Tomograph (General Electric, Milwaukee, WI, USA) in 3D mode. The patients in this cohort underwent PET scanning in the STN DBS “OFF” state (as above, the absence of STN stimulation for at least 2 h prior to PET scanning). Following a transmission scan, a PET image was acquired over 45–55 min after an intravenous injection of ^18^F-FDG (8–10 mCi) and was reconstructed with the 3D reprojection method. All subjects were scanned in a resting state with the eyes open in a quiet and dimly lit room.

Preprocessing of brain PET images from each participant was performed using Statistical Parametric Mapping software (SPM8) implemented in MATLAB 9.1.0 (MathWorks Inc, Sherborn, MA). The images were spatially normalized into the Montreal Neurological Institute (MNI) brain space with linear and nonlinear 3D transformations. The normalized PET images were then smoothed by a Gaussian filter of 8-mm FWHM over a 3D space to increase the signal-to-noise ratio for statistical analysis. Local metabolic activity normalized to global activity was measured in 95 regions of interest (ROIs). In addition to 90 ROIs from the automated anatomical labeling (AAL) atlas [19], this analysis included 5 discrete ROIs corresponding to the vermis, bilateral cerebellum (excluding the vermis) and bilateral pons [7].

### Pattern analysis

To identify a significant PDRP topography, we applied the Scaled Subprofile Model and Principal Component Analysis (SSM/PCA) to ^18^F-FDG PET images from combined PD1 patients and HC1 subjects in cohort I using Scan Analysis and Visualization Processor (ScAnVP software V6.0, software package available on https://www.feinsteinneuroscience.org at the Center for Neuroscience, Feinstein Institute for Medical Research, NY) [20]. This analysis was done through a regional approach analogous to that described previously, in which subject × voxel covariance analysis of group-level image data was applied to measurements of metabolic activity using an automated computerized algorithm [21, 22]. The PDRP pattern was acquired by the first principal component (PC1), whose expression could discriminate PD patients from healthy subjects. The reliability of voxel weights on the PDRP pattern was assessed using bootstrap resampling described previously [23]. The procedure entails performing the SSM/PCA method 100 times on PET data that were resampled with replacement from the pool of PD and HC subjects. Next, the Z-score of each voxel was calculated, and the threshold of 3.35 was used to measure the reliability of voxel weight. The expression of the PDRP pattern of each subject was calculated using the topographic profile rating (TPR) algorithm and represented by a Z-transformed score using scores of the HC1 subjects [6]. The DBS treatment effects of PD patients were represented predominantly by PDRP expression changes. These expression scores were also Z-transformed using scores of HC1 subjects and subtracting the offset value of HC2 to eliminate the effects of different scanning devices.

The derived metabolic topography was further assessed by an ROI-based pattern of regional weights in a set of salient abnormal brain regions. Therefore, we measured the PDRP regional weights and identified the salient abnormal regions. In brief, the PDRP regional weights were derived by the mean standardized voxel weights within an ROI and then Z-transformed based on the mean and standard deviation of 95 predefined ROIs. We then divided the brain into disease subspace and non-subspace based upon the ROI-wise weights, corresponding, respectively, to ROIs with high (absolution region weight greater than or equal to 1) and low (absolution region weight below 1) local contributions to the overall PDRP pattern [15].

### Graph theory-based brain connectome analysis

To better understand the pattern variation within PDRP concurrent with STN DBS treatments over a relatively long time period, graph theory-based brain connectome analysis was implemented for cohort II. This analysis has been widely applied to reveal the changes of local neural functional connectivity in neurodegenerative diseases [12–14, 24]. In this study, the ^18^F-FDG PET image signals in ROIs reflected metabolic synaptic activity. A graphical network representation of inter-ROI coherence was constructed. Network nodes in each network were defined as the 95 standardized predefined ROIs. Network connectivity was defined as metabolic correlations between pairs of network nodes that denote interneuronal information transfer [25]. The metabolic activity in each of the ROIs was used to generate a region × region correlation matrix (95 × 95 for PD2 and HC2 groups). The correlation matrix based on absolute values of pairwise regional correlation coefficients was constructed for each group by Pearson correlation. Then, the adjacency matrices (binary, undirected matrices) were generated from the absolute correlation matrix at varying sparsity thresholds (1–50%, 1% as the threshold interval), where the sparsity threshold is defined as the connection density, which is the ratio of existing connections to possible connections [15]. We calculated the minimum sparsity threshold of fully connected data for the PD2 and HC2 groups.

For all experimental groups, we calculated three network properties as follows: (1) the clustering coefficient (*C*), which is a measure quantifying the degree to which nodes in a network tend to cluster together and is a representation of network segregation, measuring the local information transmission capability in a network [26]; (2) the characteristic path length (*L*), which is a measure of network efficiency defined as the average shortest path length on the graph, measuring the global information transmission capability in a network; and (3) the small-worldness coefficient (*S*), which is the C/L ratio for the graph divided by the analogous measure for a random graph with the same sparsity [27]. To avoid biases related to a single threshold, the above-mentioned properties were computed at varying sparsity thresholds, stepwise by 0.01 intervals.

In our study, differences in the analytical parameters were evaluated separately for the PDRP and non-PDRP spaces of the experimental groups, as well as corresponding group × subspace interaction effects. This analysis was performed with 1000 permutations of random samples from the combined subject pool at each sparsity threshold. Graph theoretic measures were performed using the Brain Connectivity Toolbox (https://www.brain-connectivity-toolbox.net/) [28].

### Statistical analysis

Differences of PDRP expression between PD and HC subjects in each cohort were evaluated by two-sample Student’s *t* test. The changes in PDRP scores and UPDRS motor ratings were evaluated using one-way repeated-measure ANOVA (RMANOVA) with post hoc Bonferroni tests across 3 follow-up intervals. We also used paired-samples *t* tests when comparing PDRP scores and UPDRS motor ratings between the first and the second follow-up intervals. The network properties for the PDRP subgraphs and the differences between the subgraphs were compared across samples using permutation tests. All statistical analyses were performed using SPSS 22.0 software (SPSS Inc., Chicago, IL) run on a Windows platform. Values were considered significant at *P* < 0.05 with 2-tailed tests.

## Results

### PDRP identification and expression

**Fig. 1:**
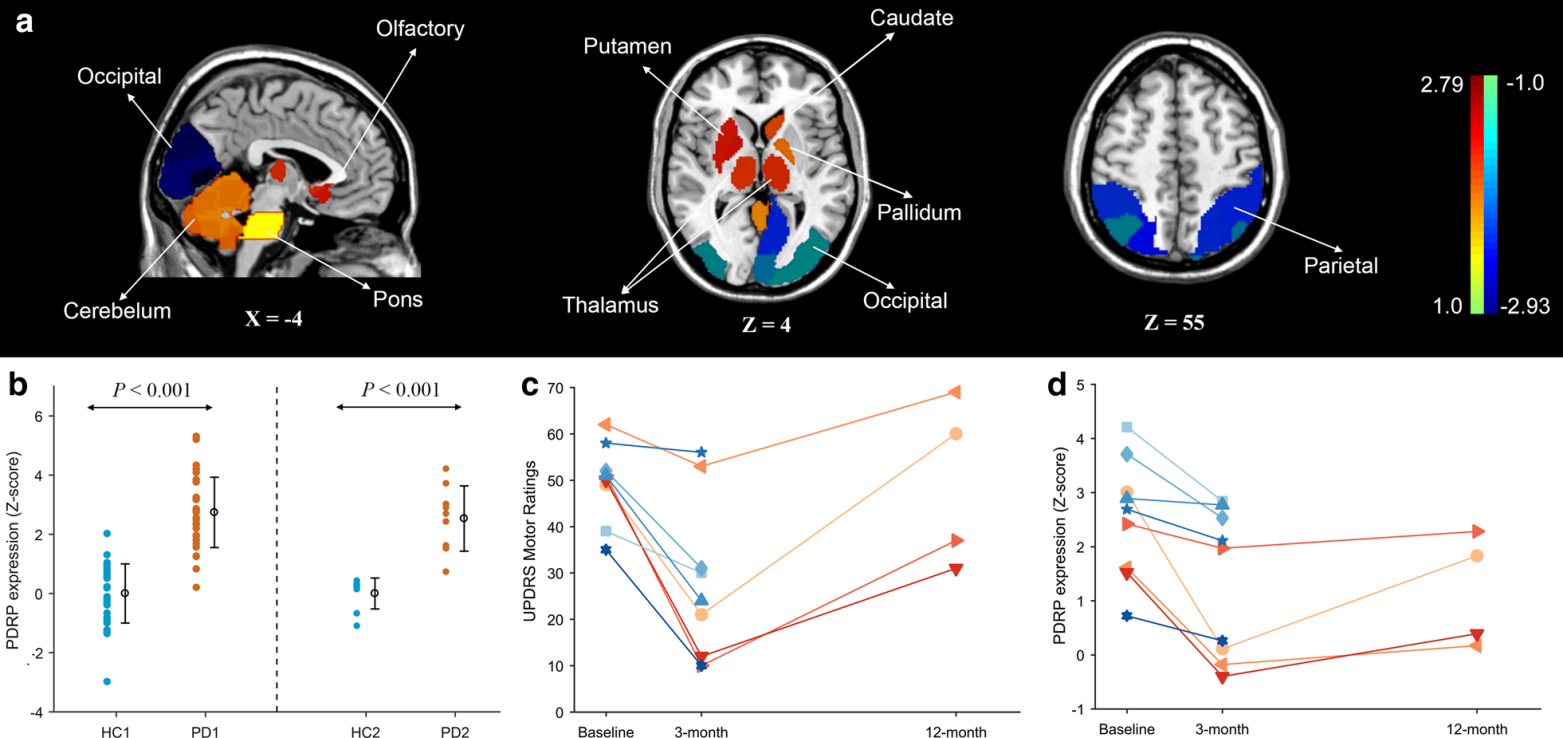
**a** PDRP identified by pattern analysis in ^18^F-FDG PET images from PD1 patients and HC1 subjects. This pattern was characterized by relatively higher metabolism in the bilateral putamen, pallidum, caudate, thalamus, cerebellum, pons and olfactory region, which was associated with decreased metabolism in the posterior parietal–occipital cortices. Regions with metabolic increases (positive region weights) are color-coded from red to yellow; those with metabolic decreases (negative region weights) are color-coded from blue to purple. **b** The expression of the PDRP pattern showed that the subject scores of PD patients were relatively higher than those of HC subjects in both cohort I and cohort II (*P* < 0.001). UPDRS motor ratings (**c**) and PDRP scores (**d**) in PD2 patients at preoperative baseline, 3-month post-operation and 12-month post-operation in STN DBS “OFF” states both exhibited significant changes across intervals (*P* < 0.01; RMANOVA). The scatters of different colors and shapes represent the PD patients with DBS treatment

Pattern analysis of individual ^18^F-FDG PET images in cohort I identified ROI-based PDRP with the first principal component, which accounted for 17.99% of subject × voxel variance. This pattern was characterized by relative metabolic increase in the putamen, pallidum, caudate, thalamus, cerebellum, pons and olfactory region, associated with metabolic decrease in the posterior parietal–occipital cortices (Fig. 1a).

The scores of PDRP expression were abnormally elevated in PD1 patients compared with HC1 subjects (*P* < 0.001; two-sample *t* tests; Fig. 1b). Similarly, prospectively computed PDRP scores of PD2 patients at preoperative baseline were also increased (*P* < 0.001; two-sample *t* tests; Fig. 1b) relative to those of HC2 subjects. For the PD2 patients, the trend in individual values for the UPDRS motor rating and PDRP expression at three timepoints (preoperative baseline, 3-month post-operation and 12-month post-operation), both exhibited significant changes in activity over time (UPDRS: *F*_(2,6)_ = 11.101, *P* = 0.010; PDRP: *F*_(2,6)_ = 10.596, *P* = 0.011; RMANOVA), are summarized in Table 1, Fig. 1c, d. Indeed, we observed a significant decrease in UPDRS (*P* = 0.028, post hoc test; *P* = 0.001, paired-sample *t* tests; Fig. 1c) and PDRP scores (*P* = 0.039, post hoc test; *P* = 0.004, paired-sample *t* tests; Fig. 1d) from baseline to the second follow-up timepoint, but a slight increase from the second to the third timepoint (UPDRS: *P* = 0.016; PDRP: *P* = 0.094, post hoc test). The changes in PDRP expression mediated by STN DBS were generally in line with improvement of UPDRS motor ratings in PD2 patients. For the UPDRS motor ratings, 2 of the 4 PD2 patients showed higher scores at the third timepoint with respect to baseline, whereas the other 2 patients had slightly lower scores at the third timepoint relative to baseline. For PDRP expression, all 4 patients showed relatively lower scores at the third timepoint compared with those at baseline.

### Graph-theory-based analysis

Graph-theory-based analysis was also used to disclose the longitudinal changes in network measures in PD patients who underwent STN DBS at different follow-up intervals. For this analysis, the whole brain was divided into 2 discrete subspaces (PDRP and non-PDRP subspaces) depending on the region weights. We highlighted PDRP subspaces with 29 ROIs, in which 13 ROIs had positive region weights that were greater than + 1.0, while the other 16 ROIs had negative region weights that were lower than − 1.0 (Additional file 1: Table S1). In all participant groups, the minimum sparsity threshold in which the network was fully connected was calculated: 10% (PD2, preoperative baseline), 25% (PD2, 3-month post-operation), 12% (PD2, 12-month post-operation) and 12% (HC2). The network properties were calculated at various sparsity thresholds in the range of 25–50% for PD2 and HC2.

**Fig. 2:**
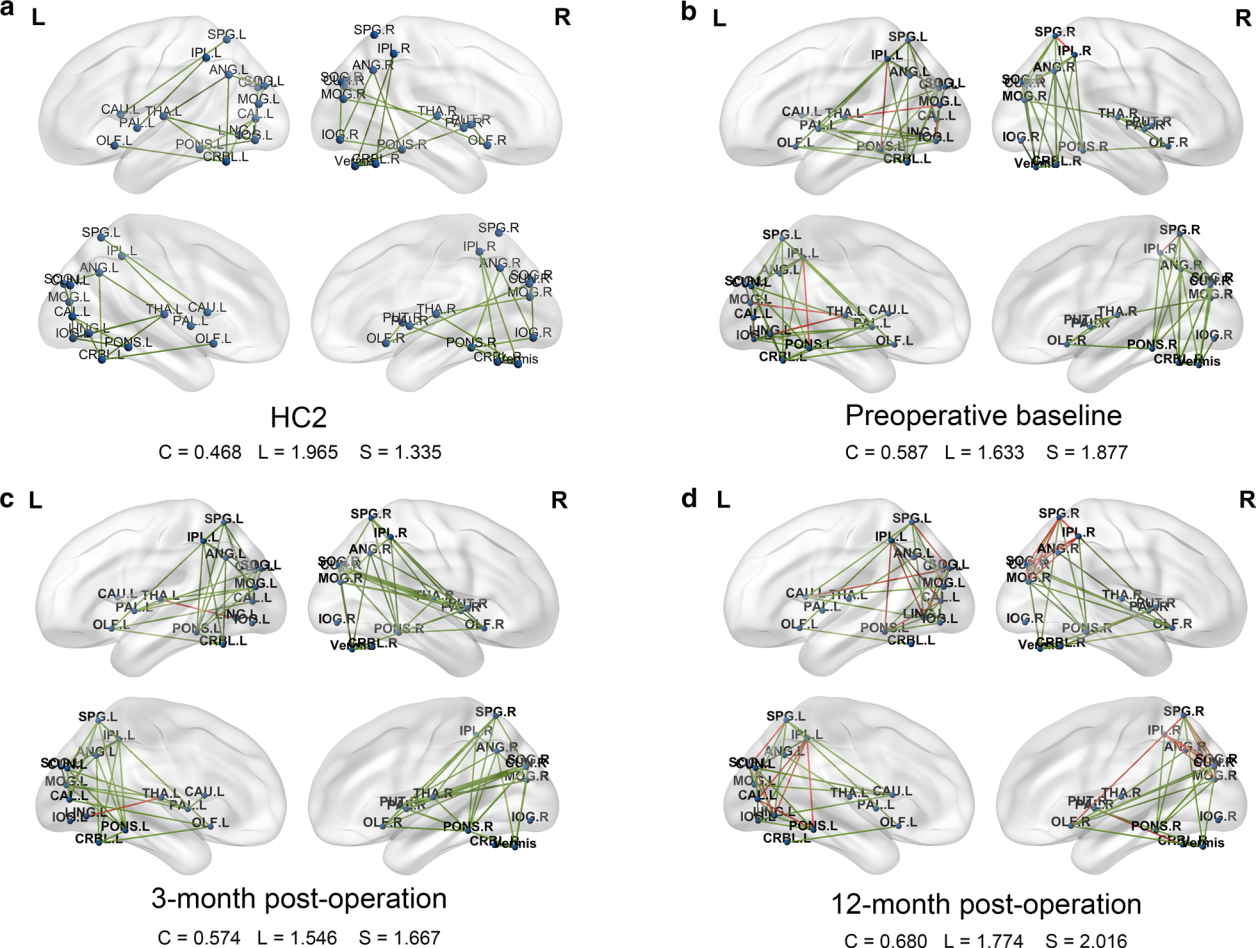
The metabolic connection network of HC2 subjects (**a**), PD2 patients at baseline (**b**), PD2 patients at 3-month post-operation (**c**), PD2 patients at 12-month post-operation (**d**) in PDRP subspaces at a sparsity threshold of 25%. The blue circle nodes indicate different anatomical brain regions. Red and green lines indicate pathological (i.e., significantly altered, *P*-value with FDR correlated < 0.05) and normal metabolic connection between brain regions of PDRP subspaces

With the analysis of the network properties for PDRP subspaces at a sparsity threshold of 25% (Fig. 2a, b), the clustering coefficient (*C*) of PD2 at the preoperative baseline increased compared with that of HC2 (HC2: *C* = 0.468; PD2-baseline: *C* = 0.587). In contrast, the characteristic path length (*L*) of PD2 at preoperative baseline decreased relative to HC2 (HC2: *L* = 1.965; PD2-baseline: *L* = 1.633), with corresponding increases in the small-worldness coefficient (*S*) (HC2: *S* = 1.335; PD2-baseline: *S* = 1.877). The difference in the PDRP subspaces between HC2 and PD2 at preoperative baseline was significant given the sparsity threshold (*P* < 0.05; 1000 permutations).

With the analysis of the network properties within PDRP subspaces for PD2 patients at 3 different follow-up intervals under a sparsity threshold of 25% (Fig. 2b–d), the small-worldness coefficient decreased from baseline to the second timepoint, but increased between the second and third timepoints (PD2-baseline: *S* = 1.877; PD2-3 months: *S* = 1.667; PD2-12 months: *S* = 2.016). Analogous measure differences were also discovered for the clustering coefficient (C) and the characteristic path length (*L*) (PD2-baseline: *C* = 0.587, *L* = 1.633; PD2-3 months: *C* = 0.574, *L* = 1.546; PD2-12 months: *C* = 0.680, *L* = 1.774). The difference in the PDRP subspaces between baseline and 3-month post-operation, and between 3-month post-operation and 12-month post-operation was all significant given the sparsity threshold (*P* < 0.05; 1000 permutations). In addition, the network properties of each group, over the entire sparsity threshold range (25–50%), are shown in Additional file 1: Fig. S1.

### Discussion

This study demonstrated metabolic network can capture the longitudinal changes of PD patients who had undergone STN DBS treatment. Clinical assessments of our cases post-operation at 3-month follow-up showed that electrical stimulation markedly improved motor function of each patient by an average 46% compared with that at baseline prior to the surgery, whereas followed by a reversal of UPDRS performance at 12-month follow-up. Such trend of UPDRS scores, which is positively correlated with changes in corresponding PDRP expression, suggesting the latter may provide a complementary biomarker for the measurement of the treatment outcome.

This study attempted to capture the individual correlation between motor performance and PDPR expression, which was proposed to represent inherent brain metabolic changes of PD patients by long-term STN DBS intervention. Significant instantaneous suppression of cerebral metabolism during effective antiparkinsonian therapies (e.g., DBS stimulation or titrated intravenous levodopa infusion) has been reported previously [10]. Although the investigation of instantaneous effect during intervention is important to understand the mechanism of the external stimuli on brain function, the ultimate treatment effects may not be revealed due to the suppression of instantaneous effect. Therefore, this longitudinal study investigated PET images in the “OFF” state instead of the “ON” state of the treatment. The PET scans were acquired at least 2 h after the STN stimulation. This allowed us to observe more stable treatment effect rather than instantaneous changes during the STN stimulations.

The strong correlation between PDRP expression and measures of the motor disability rating scale was coincidentally reported in previous studies [13, 29]. Thus, it is not surprising that changes of pattern expression were observed in general consistent with corresponding UPDRS motor ratings in this longitudinal study. Particularly, we found that pattern scores in subjects who underwent STN DBS were significantly downward modulated from baseline to 3-month post-operation. This finding agreed with the results of previous trials, showing that stimulation intervention (targeting at STN, GPi and PPN) produced marked suppression of PDRP activities in advanced PD. Similar changes in PDRP expression have also been observed during levodopa infusion as well as STN lesioning surgery as a reflection of therapeutic amelioration [30, 31]. During the longer follow-up, the PDRP expression, while kept pace with motor scores, regrettably rebounded, suggesting that STN DBS may exert the best corrective effect in a relative short-term post-operation. Although the changes of PDRP expression generally correlate with the changes of UPDRS score, we noted the trajectories of the two indices revealed some difference, with greater rollback in UPDRS motor ratings compared to corresponding PDRP expression in the same patients. It is conceivable that the relatively modest fluctuation in PDRP expression observed in this study is a reflection of cerebral modulation of motor function. Longitudinal stimulation of STN may not show a continuously significant suppression on symptomatic motor signs but instead exerts its benefit by inducing the reinforcement of motor circuit connecting the STN to subcotical and cortical motor regions. In this way, PDRP as an objective criteria may provide complimentary insight for treatment monitoring.

Graph theory also provided network-level insights into the flow of information through the PDRP subspaces. We observed PD patients at baseline with greater clustering and lower path length within the context of PDRP subspaces, giving rise to an increase in the small-world coefficient compared with the corresponding value for healthy subjects. An analogous elevation in small-worldness was previously delineated in a separate ^18^F-FDG PET study that involved a similar cohort of clinically verified PD subjects; for the first time, this study proposed an association between the exaggerated small-worldness underlying PD and the high metabolic costs and inefficiency, as well as noisy information transfer among PDRP subspaces [13, 15]. Our results also showed PDRP subspaces with decreased small-worldness coefficient toward normal at 3 months, suggesting STN-DBS stimulation tended to exert a normalizing effect that could minimize the resources required for effective information processing and communication within the PDRP subspaces in the early stage after DBS treatment. These changes were in agreement with the results of previous studies showing the beneficial effects of levodopa in partially correcting small-worldness in PDRP in individual patients [15]. Nonetheless, we also noted a significant rebound in this measure in the subsequent follow-up period, with even higher values of 12-month post-operation compared with the preoperative baseline. This variation tendency was consistent with the changes in PDRP expression over time, as mentioned above, and further indicated that DBS may exert a relatively short-term corrective effect on functional connectivity in PD brains rather than a long-term impact.

Furthermore, conventional ROI standard uptake value ratio (SUVR) analysis underlying PDRP subspaces is also calculated in Additional file 1: Table S2. The quantification of SUVR and PDRP scores actually portrayed different but complementary measures of brain dysfunction, with the former emphasizing on regional analysis and the latter tending to be a network analysis. Thus, the applications of both quantitative indexes may provide more valuable information to describe changes of brain function induced by treatment at both regional and global levels.

Several limitations of this study should be noted: (1) In the absence of a neuropathological confirmation of PD in our patient cohorts, we relied on the clinical diagnostic accuracy of movement disorder experts. This decision was a valuable strategy that reflected how PD is diagnosed in the real world. (2) The sample sizes of PD patients who underwent STN DBS in the current study were relatively small. These limited samples might be justifiable, given that this is a pilot study to first investigate longitudinal changes in whole brain network activities associated with PD patients who underwent STN DBS. Nonetheless, the limited sample size may result in sufficient statistical power. It will be necessary to replicate the results reported here in similar patients with larger sample sizes. (3) The relationship between brain glucose metabolism and clinical measures of treatment outcomes more than 1 year after DBS surgery has not been fully demonstrated due to the limited length of follow-up. While we observed the best performance in network measures in individual patients at the 3-month rather than the 12-month post-operation timepoint, we estimated a probable declined treatment effect of DBS stimulation as disease evolved by the asymmetry of DBS lead localization. (4) This study only investigated the longitudinal changes of PD patients with STN DBS “OFF” stage; future study will put more attention to the results of STN DBS “ON” stage.

### Conclusion

This study confirmed significant correlation between PDRP expression and UPDRS motor ratings by long-term STN DBS stimulation in PD patients. Reduced metabolic activity of brain network, in consistent with UPDRS motor function, was significant at the 3-month post-operation timepoint relative to baseline, but with less dramatic effects at the 12-month post-operation, indicating the short-term effect produced by STN DBS better than long-term effect. Taken together, our results demonstrate the potential of PDRP expression as a complimentary objective biomarker for the individual assessment and monitoring of STN DBS treatment outcome for PD.

## Supplementary information

**Additional file 1**. The region weights (Z-score) of PDRP subnetwork, SUVR changes of PDRP subnetwork, and the brain network properties of cohort II in the sparsity threshold 25–50% are detailed in the Additional file.

## Data Availability

All experimental data are available upon request to any of the authors of the manuscript.

## Abbreviations

PD: Parkinson’s disease
DBS: Deep brain stimulation
STN: Subthalamic nucleus
UPDRS: Unified Parkinson's Disease Rating Scale
FDG: Fluorodeoxyglucose
PET: Positron emission tomography
PDRP: PD-related metabolic covariance patterns
CSPTC: Cortico-striato-pallido-thalamo-cortical
HC: Healthy controls
MRI: Magnetic resonance imaging
MNI: Montreal Neurological Institute
ROI: Regions of interest
AAL: Automated anatomical labeling
SSM/PCA: The Scaled Subprofile Model and Principal Component Analysis
TPR: Topographic profile rating
