# Supplementary material for: Metabolic network as an objective biomarker in monitoring deep brain stimulation for Parkinson’s disease: a longitudinal study
Source: EJNMMI Res. 2020 Oct 29;10:131. doi: 10.1186/s13550-020-00722-1 (PMC7596139; doi:10.1186/s13550-020-00722-1)
Supplement: Supplementary file 1 — Additional file 1. The region weights (Z-score) of PDRP subnetwork, SUVR changes of PDRP subnetwork, and the brain network properties of cohort II in the sparsity threshold 25–50% are detailed in the Additional file. [file 13550_2020_722_MOESM1_ESM.docx]

# Additional file 1

Table S1. The region weights (Z-score) of PDRP subnetwork.

| PDRP subnetwork region | | | | | |
| --- | --- | --- | --- | --- | --- |
| *Underactive PDRP region* | | | *Active PDRP region* | | |
| Brodmann area | AAL region | Z-value | Brodmann area | AAL region | Z-value |
| *43* | Calcarine_L | -1.765 | *21* | Olfactory_L | 1.097 |
| *45* | Cuneus_L | -1.559 | *22* | Olfactory_R | 1.031 |
| *46* | Cuneus_R | -1.483 | *71* | Caudate_L | 1.431 |
| *47* | Lingual_L | -1.222 | *74* | Putamen_R | 1.121 |
| *49* | Occipital_Sup_L | -1.817 | *75* | Pallidum_L | 1.699 |
| *50* | Occipital_Sup_R | -1.803 | *76* | Pallidum_R | 1.081 |
| *51* | Occipital_Mid_L | -1.959 | *77* | Thalamus_L | 1.245 |
| *52* | Occipital_Mid_R | -1.929 | *78* | Thalamus_R | 1.288 |
| *53* | Occipital_Inf_L | -1.811 | *91* | Cerebelum_L | 1.611 |
| *54* | Occipital_Inf_R | -2.040 | *92* | Cerebelum_R | 1.441 |
| *59* | Parietal_Sup_L | -1.269 | *93* | Vermis | 1.841 |
| *60* | Parietal_Sup_R | -1.055 | *94* | Pons_L | 2.597 |
| *61* | Parietal_Inf_L | -1.220 | *95* | Pons_R | 2.724 |
| *62* | Parietal_Inf_R | -1.298 |  |  |  |
| *65* | Angular_L | -1.814 |  |  |  |
| *66* | Angular_R | -1.885 |  |  |  |

Table S2. SUVR changes of PDRP subnetwork.

| Brain region | SUVR change between 3-month follow-up and baseline | SUVR change between 12-month and 3-month follow-up |
| --- | --- | --- |
| Caudate_L | -6.02±4.14%** | 3.06±4.31% |
| Putamen_R | -3.92±3.47%** | 7.19±3.04%* |
| Pallidum_L | -2.99±5.10% | 4.18±2.71%* |
| Pallidum_R | -3.91±3.63%* | 7.82±5.38% |
| Thalamus_L | -3.70±3.74%* | 3.13±3.03% |
| Thalamus_R | -5.31±3.18%** | 4.36±4.39% |
| Cerebelum_L | -0.81±4.36% | 2.73±6.22% |
| Cerebelum_R | -3.47±4.66% | 4.27±4.58% |
| Vermis | -3.51±4.09%* | 4.81±4.01% |
| Pons_L | -7.42±6.43%** | 7.34±9.42% |
| Pons_R | -8.07±5.92%** | 9.68±10.4% |
| Olfactory_L | -3.51±3.97%* | 0.34±5.82% |
| Olfactory_R | -3.19±4.21%* | 1.37±4.06% |
| Calcarine_L | 3.52±3.25%* | -1.61±2.73% |
| Cuneus_L | 3.38±4.65% | -1.88±2.43% |
| Cuneus_R | 3.15±3.94%* | -1.44±2.58% |
| Lingual_L | 1.71±2.17%* | -0.66±3.74% |
| Occipital_Sup_L | 2.93±5.23% | -2.51±3.42% |
| Occipital_Sup_R | 2.87±4.36% | -2.35±1.88%* |
| Occipital_Mid_L | 2.63±3.74% | -4.53±1.23%** |
| Occipital_Mid_R | 1.81±2.85% | -3.74±0.68%** |
| Occipital_Inf_L | 2.64±3.45%* | -1.07±6.59% |
| Occipital_Inf_R | 1.26±3.48% | -2.14±2.75% |
| Parietal_Sup_L | 4.95±4.48%* | 0.29±4.76% |
| Parietal_Sup_R | 4.27±4.79%* | 0.73±3.47% |
| Parietal_Inf_L | 3.88±3.03%** | -0.09±4.62% |
| Parietal_Inf_R | 3.21±4.45% | 0.51±2.74% |
| Angular_L | 3.57±4.73% | -2.45±1.52%* |
| Angular_R | 3.47±6.04% | -3.03±4.75% |

Note: SUVR change = (SUVR of follow-up – SUVR of baseline)/SUVR of baseline * 100%; SUVR is calculated using global mean uptake as reference value.

* is *P* < 0.05; ** is *P* < 0.01; *P* value is derived by one sample t-test for mean equal to zero.


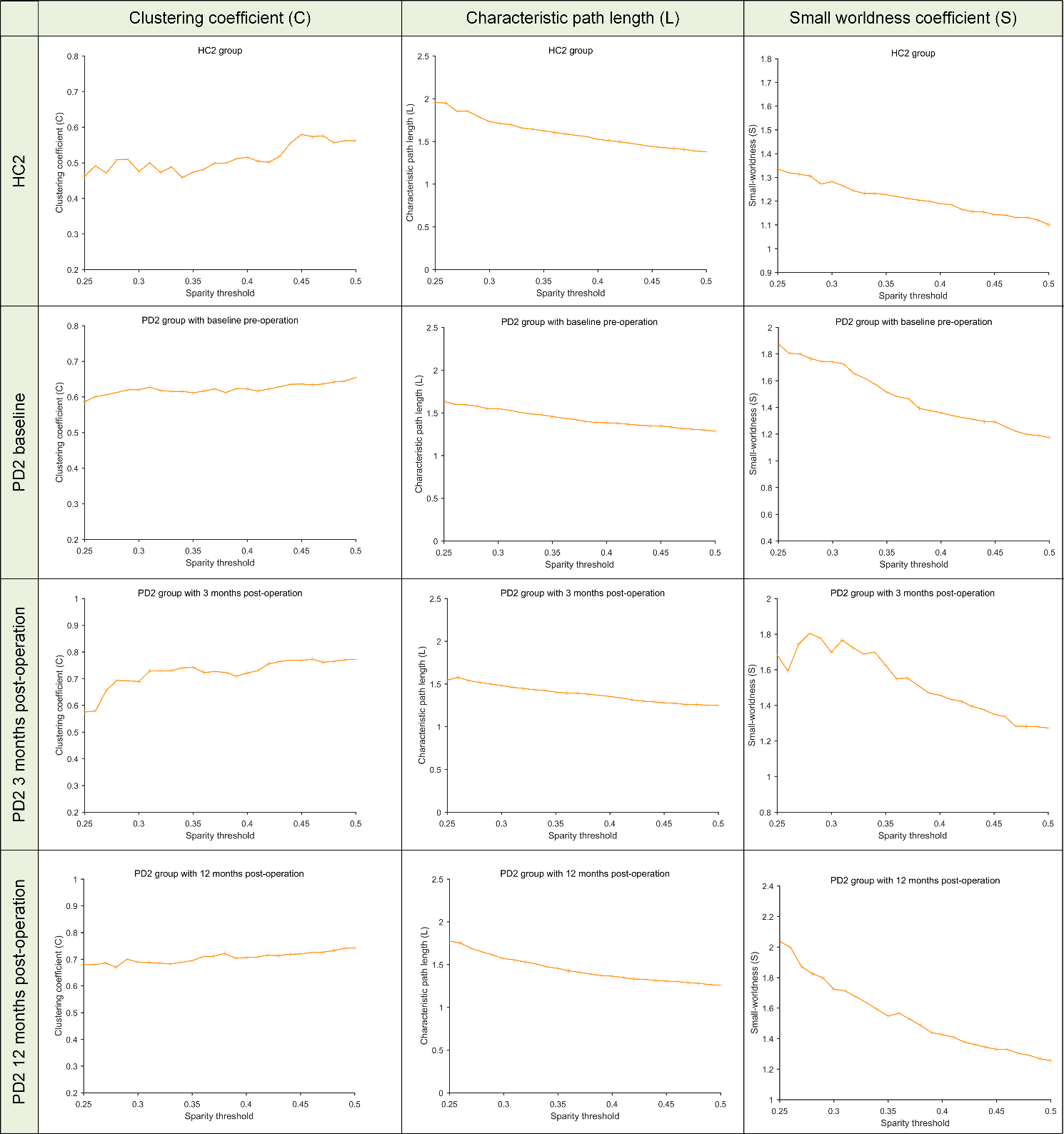


**Fig. S1.** The brain network properties of cohort II in the sparsity threshold 25%-50%.
